# Ion-selective asymmetric carbon electrodes for enhanced capacitive deionization†

**DOI:** 10.1039/c7ra10443j

**Published:** 2018-01-11

**Authors:** Tingting Yan, Baoxia Xu, Jianping Zhang, Liyi Shi, Dengsong Zhang

**Affiliations:** Research Center of Nano Science and Technology, Shanghai University Shanghai 200444 China dszhang@shu.edu.cn +86 21 66136079

## Abstract

With the development of capacitive deionization technology, charge efficiency and electrosorption capacity have become some of the biggest technical bottlenecks. Asymmetric activated carbon electrodes with ion-selective functional groups inspired by membrane capacitive deionization were developed to conquer these issues. The deionization capacity increased from 11.0 mg g^−1^ to 23.2 mg g^−1^, and the charge efficiency increased from 0.54 to 0.84, due to ion-selective functional groups minimizing the co-ion effect. The charge efficiency and electrosorption capacity resulting from better wettability of these electrodes are effectively enhanced by grafting ion-selective functional groups, which are propitious to ion movement. In addition, asymmetric deionization capacitors show better cycling stability and higher desalination rates. These experimental results have demonstrated that the modification of the ion-selective (oxygen-containing) functional groups on the surfaces of activated carbon could greatly minimize the co-ion effects and increase the salt removal from the solution. These results have indicated that the ion-selective asymmetric carbon electrodes can promote well the development of deionization capacitors for practical desalination.

## Abstract 2

Ion-selective asymmetric carbon electrodes are developed for capacitive deionization to minimize the co-ion effects.

## Graphical abstract

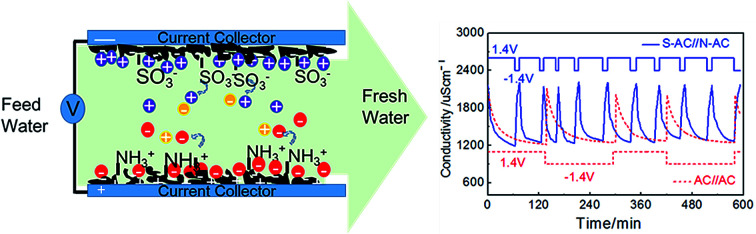

## 1. Introduction

The water crisis is one of the most threatening issues in the foreseeable future due to the decrease of available fresh water caused by the growing population and environmental pollution.^1–4^ Deionization of brackish water can provide abundant fresh water for humans. Various desalination technologies have been developed to conquer this threat.^1,3,5,6^ Capacitive deionization (CDI) as a new technology is more energy-efficient compared to such traditional desalination approaches as reverses osmosis and multistage distillation.^7–13^ The cardinal principle of CDI is the same as for electric double layer capacitors (EDLCs), but with distinct differences.^14–16^ The main difference is that the solution is fluid and ions are removed and stored on the electrodes with the applied voltage (1–2 V) during the deionization process, whereas the electrolyte is immovable in EDLCs and is only for energy storage.^14,17,18^ The goal of CDI is the removal of ions from the electrolyte rather than energy storage.^16^

According to the principle of CDI, carbon materials are more suitable due to their unique properties. Hence, various carbon materials have been developed by us and other research groups.^19–22^ Most of them show good desalination performance but prepared with a complex procedure, high cost and low-scale yield.^23,24^ Among them, activated carbon (AC) has been recognized as the most commercially practical electrode material for CDI due to its higher specific surface area, larger pore volume, better stability, lower cost and mass production.^25–28^ Unfortunately, AC has high surface area but always accompanied with a large amount of disordered arrangement of micropores, which restrict the diffusion of salt ions and mass transfer in desalination process.^25,28,29^ Although advanced strategies were proposed to solve this issue including increasing the ratio of mesoporous structure and introducing some hydrophilic groups on the AC electrodes.^25,30^ The electrosorption capacity and charge efficiency are still much lower than that expected due to the co-ion expulsion effects.

Although the CDI technology has many advantages, the co-ion expulsion effect is an unavoidable issue, in which the oppositely charged counter-ions are adsorbed to the electrode and co-ions are repelled by applying voltage to electrodes.^2,31^ The adsorption and desorption of salt ions occur at the same time in the deionization process, which result in reducing the charge efficiency (CE, defined as the ratio of adsorbed salt over charge) and increasing the energy consumption.^10^ Generally, higher charge efficiency leads to lower energy consumption. However, the charge efficiency of most carbon electrodes in the CDI process is lower than 0.6, which is far less than 1 and limits its large-scale industrial application.^2,4,10^ To promote the practical application of CDI technology, it is quite urgent to improve the charge efficiency and reducing energy consumption of the electrodes.

These limitations may be overcome effectively by introducing ion-exchange membranes (IEM) into the CDI.^7,32^ The ion-exchange membranes capacitive deionization (MCDI) have ion selectivity which prevents reverse adsorption and prohibits the mobility of co-ions.^33^ The movement of counter-ions is free and the co-ions are prohibited in the IEM.^34^ It will minimize the co-ions expulsion effect and increase CE and salt removal efficiency. It has been demonstrated that the CE of MCDI or revised-MCDI is even up to 0.9.^32^ However, there are two disadvantages limiting the commercial application of MCDI. One is that the price of IEM is very high and the other is the high contact resistance caused by the inferior contact adhesion between the CDI electrodes and IEM.^31^ The electrode with grafting ion-selective groups through covalent bonds is analogous to the MCDI. It can simplify the equipment and overcome the disadvantages of MCDI, which is more economical. Inspired by the MCDI, carbon electrodes with grafting ion-selective groups have attracted great interest of the CDI technology.^2,31,35–37^ Previously, we designed and prepared a novel ion-selective 3D graphene electrode to overcome the co-ions expulsion effect.^31^ However, the construction of ion-selective 3D graphene suffered from high production cost, long-time treatment and only suitable for lab-scale fabrication.^31^ It is noted that most of asymmetric CDI were designed and prepared to overcome the co-ions expulsion effects, in which only one pair of asymmetric electrode were studied in a low concentration and small volume of NaCl solution for laboratory research.^31,35–37^ The CDI for practical applications is still challenging.

**Fig. 1:**
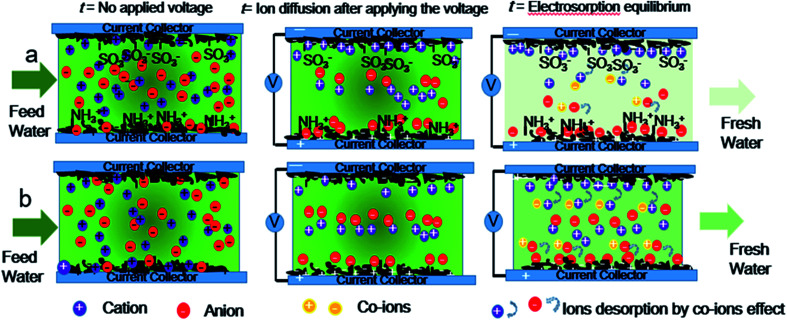
Scheme illustration of the time-dependent CDI model of (a) asymmetric AC electrodes and (b) symmetric activated carbon electrodes.

Hence, asymmetric AC electrodes have been designed and grafted by sulfonic groups and amine groups on the surface of AC through covalent bonds as a cation-selective electrode and an anion-selective electrode respectively for enhanced capacitive deionization. The NaCl aqueous solution with an initial concentration of 1000 mg L^−1^ in a total volume of 400 mL was pumped to the cell from beginning to end in this work. Ten pairs of asymmetric electrodes are assembled with sulfonic AC as positive electrodes and aminated AC as negative electrodes. The scheme of co-ion minimization is illustrated in Fig. 1. The ions are selectively absorbed on the surface of asymmetric AC electrodes even before applying the voltage. Ions flux into and store in the oppositely charged electrode pores with electrostatic attraction after applying voltage. Ion-selective functional groups can prevent reverse adsorption and prohibit the mobility of co-ions similar to MCDI. As a result, the electrosorption capacity and charge efficiency of asymmetric AC electrodes have been significantly improved because of not only preventing co-ion expulsion effect but also promoting the wettability and accelerating salt solution infiltration. These results will be beneficial to solve the technical bottlenecks and accelerate the practical engineering application of CDI.

## 2. Experimental details

### Preparation

AC (TF-B518) was supplied by Shanghai Sino Tech Investment Management Co. Ltd and modified with diluted nitric acid. Sinopharm Chemical Reagent Company provided other chemicals. DI water was used in the whole experiment process.

The preparation of ion-selective function AC was according to the literature with some improvements.^31^ For anion groups grafting, 10.5 g of sulfanilic acid was dissolved to the NaOH aqueous solution in an ice bath. 65 mL of icy hydrochloric acid was added to the above solution for 15 min. Then, 30 mL of sodium nitrite was added to the above solution slowly for 30 min. Next, the diazosalt of sulfanilic acid was obtained and added slowly to the AC dispersion for 3 h. The products were collected by centrifugation, washed several times with deionized water and absolute ethanol, and dried at 60 °C for 12 h. The obtained sample was labelled as S-AC. For cation groups grafting, 2.5 mL of 3-aminopropyltriethoxysilane was dispersed to 1000 mL of acetone dispersion of AC and then evaporate all the acetone at about 70 °C. The obtained samples was labelled as N-AC.

#### The corresponding electrodes preparation

The carbon ingredient and PVDF were uniformly mixed in *N*-methyl pyrrolidone with ratio of 85 : 15 in a vacuum mixer. Then the mixture was pressed onto graphite sheets with a mass of 1.0 g and a size of 7.0 cm × 11.0 cm × 0.01 cm. The corresponding electrodes are obtained after dried at 40 °C overnight.

### Characterization

The samples were characterized by nitrogen sorption isotherms (Autosorb-IQ2, Quantachrome Corporation), field emission scanning electron microscopy (SEM, JEOL JSM-700F), X-ray photoelectron spectroscopy (XPS, Perkin-Elmer PHI 5000C ESCA). Fourier transform infrared spectroscopy (FT-IR, Thermo Nicolet Avatar 370 spectrometer) and dynamic contact angle analysis (Krüss, DSA100). The detailed information is available in ESI.†

### Electrochemical and desalination experiments

Cyclic voltammetry (CV) was actualized in a 3-electrode system using a CHI 660D, which include an S-AC (or N-AC or AC) as the working electrode, a graphite sheet as the counter electrode, and a saturated calomel electrode as the working electrode.

The electrosoption performance was conducted in electrosorption cell. The electrosorptive cell includes 10 pairs of electrodes and each pair electrodes separated by an inert spacer. Four different CDI cells were assembled for comparison: (1) AC *versus* N-AC, which is cathode and anode correspondingly; (2) AC *versus* S-AC, which is anode and cathode respectively; (3) N-AC *versus* S-AC, where is anode and cathode correspondingly; (4) AC *versus* AC, which is symmetric combination. Aqueous sodium chloride solution with fresh concentration of 1000 mg L^−1^ and total volume of 400 mL was pumped to the cell from beginning to end. The conductivity of NaCl solution was detected by a conductivity meter to reflect the concentration changes.

## 3. Results and discussion

### Characteristics

**Fig. 2:**
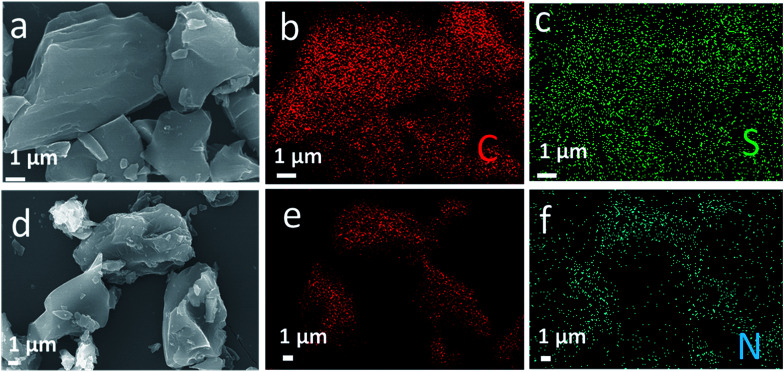
(a) SEM images of S-AC and (d) NS-AC, (b and c) EDS elemental mapping of C, S and (e and f) C, N correspondingly.

The surface profile of the obtained materials is determined by SEM images and shown in Fig. 2. As seen from Fig. 2a and d, both S-AC and N-AC exhibit well connected and irregular network-like porous architectures. It indicates that the morphology and structure of AC are mainly maintained after modification. The EDS mapping (Fig. 2b–f) confirms that S and N element are uniformly scattered on the whole surface of the S-AC and N-AC correspondingly. It well proved that the ion-selective groups are effectively grafted on the surface of AC, which should be beneficial to electrosorption.

**Fig. 3:**
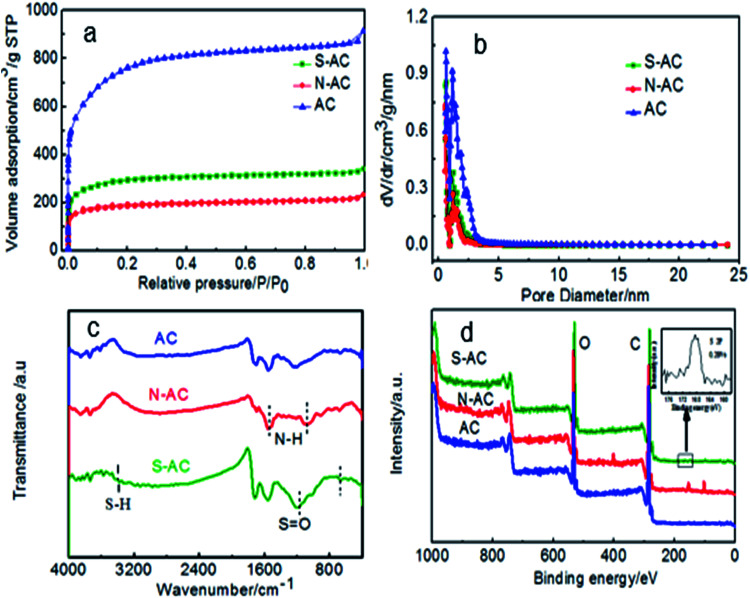
(a) Nitrogen adsorption/desorption isotherms, (b) pore size distribution, (c) FTIR spectra and (d) XPS spectra (the insert is the S 2p spectra.) of AC, S-AC and N-AC.

Pore features and specific surface area are represented by N_2_ sorption isotherms (Fig. 3a and b). Generally, the surface area of AC includes micropore surface area and the external surface area.^29^Fig. 3a shows the N_2_ adsorption isothermal of AC, N-AC and S-AC. According to the IUPAC classification, all the samples exhibit a typical type I, which indicate that the presence of relatively large micropore in the frameworks.^35–37^ The BET specific area decreased from 2759 m^2^ g^−1^ for AC to 1090 m^2^ g^−1^ for S-AC and 696 m^2^ g^−1^ for N-AC, especially, the specific area of N-AC is the lowest because the grafted groups may increase the total weight of the samples and decrease the specific surface area correspondingly. Although the decrease of the specific surface area of S-AC and N-AC, the wettability of S-AC and N-AC are greatly increased due to the hydrophilic –SO_3_^−^ and –NH_3_^+^groups on the surface, which can result in full contact between the salt solution and electrodes, accelerate salt ion infiltration, and enhance the deionization performance.^2,31^

The presence of ion-selective functional groups of the AC, N-AC and S-NC are confirmed by the FT-IR spectra (Fig. 3c). The peaks at around 3500–3400 cm^−1^ and 1750–1600 cm^−1^ exist in all the samples, which are assigned to O–H stretching and CO asymmetric stretching vibration, respectively. The characteristic peaks at around 1150–1050 cm^−1^ and 650–575 cm^−1^ in the sample S-AC are ascribed to the symmetric stretching vibration of S=O, indicating effective grafting of sulfonic groups on the surface of S-AC. The peak at around 1633 cm^−1^ and 1123 cm^−1^ corresponding to bending vibration of N–H and stretching vibration of C–N are obviously observed indicating the combination of –NH_2_ groups on the N-AC. The presence of the characteristic peaks of –NH_2_ and S=O demonstrate that sulfonic and amine groups have been successfully decorated on the surface of S-AC and N-AC.

The surface compositions of asymmetric AC was detected by XPS. As displayed in Fig. 3d, the N-AC sample shows an obvious peak at ∼400 eV assigned to N 1s, and the atomic percentage of N element was about 4.39%. Other samples shows no obvious N 1s peak. The S-AC sample shows a notable peak at 167.5 eV of S 2p. From the inset picture, the atomic percentage of S element was about 0.28%. In addition, no obvious S 2p peak was detected even after the signal amplification for others. All the results can prove that the ion selective groups have been successfully grafted on AC.

**Fig. 4:**
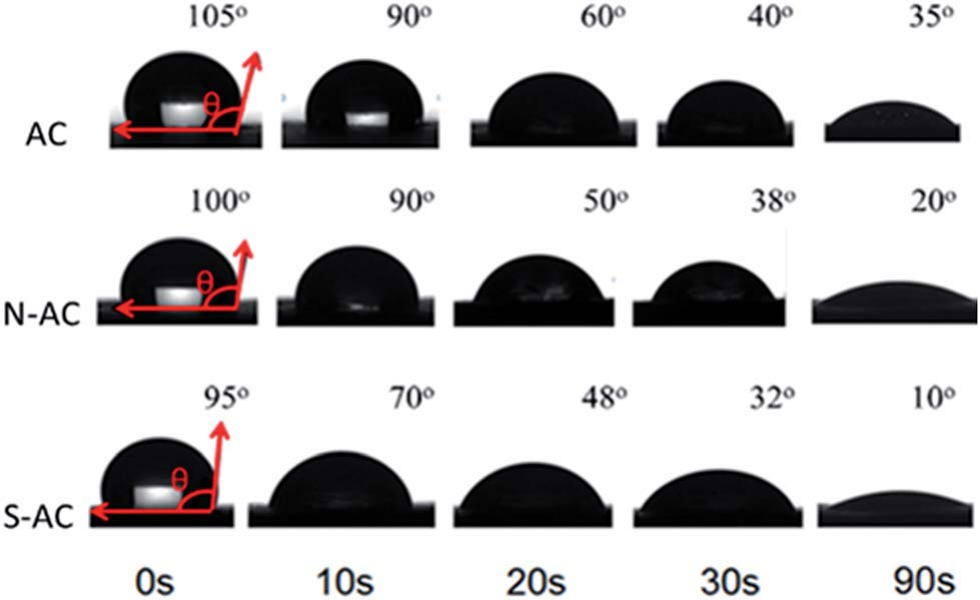
Optical micrographs of the water contact angles of AC, S-AC and N-AC at different time.

The dynamic contact angle measurements are carried out to better understand the function of modification on the wettability. The contact angle changes over time as presented in Fig. 4. The contact angle of AC (105°) is higher than N-AC (100°) and S-AC (95°) when water drops on the electrode surfaces as soon as possible. The contact angle of the N-AC and S-AC are decreased faster with time increasing. Then, contact angles of AC, S-AC and N-AC are down to 35°, 10°, and 20° at the contact time about 90 s. It should be pointed out that N-AC and S-AC have lower contact angle, which indicates that the hydrophilic –SO_3_^−^ and –NH_3_^+^groups on the surface increase the electrode's wettability. It is consistent with the above XPS and FT-IR results.

### Electrochemical properties

The CV was usually employed to evaluate the electrosorption ability of electrodes.^38^ All the CV curves show typical capacitor-like characteristics with no obvious oxidation/reduction peaks in the selected voltage range (Fig. S1†). It manifests that the capacitive behavior of all samples result from EDLC assigning to the coulombic interactions.^39^ But the shape of CV curves is slightly deviated from rectangle because of the inherent resistivity of the salt solutions.^40^

Generally, the larger area of CV curves indicates higher specific capacitance under the same condition. It needs to note that the CV curve of N-AC and S-AC electrodes exhibit much larger area as compared with that of AC, suggesting a higher specific capacitance of N-AC and S-AC electrodes. The specific capacitances of AC, N-AC and S-AC electrodes are 39.3 Fg^−1^, 44.6 Fg^−1^, and 57.9 Fg^−1^ according to the eqn (S1),† respectively. The enhanced capacitances can be attributed to the grafted –SO_3_^−^ and –NH_3_^+^ groups. The CV curves at 5 mV s^−1^ are also carried out (Fig. S1b†). It can be seen that the shape of CV curves are relatively rectangular. Generally, the lower scan rate, the higher specific capacitance. The CV curves at 10 mV s^−1^ in a 1000 mg L^−1^ NaCl solution are seriously deviated from the relatively rectangular shape (Fig. S1c†). The area of the CV profiles are smaller, indicating a lower specific capacitance.

### Desalination performance

In order to investigate the influence of ion-selective groups of asymmetric activated carbon on deionization performance, four sets of different CDI cells were evaluated for comparison. Four different enhanced CDI cells include three sets of asymmetric electrodes and one set of symmetric electrodes: (1) AC *versus* N-AC, which is cathode and anode correspondingly; (2) AC *versus* S-AC, which is anode and cathode correspondingly; (3) N-AC *versus* S-AC, which is anode and cathode correspondingly. As mentioned above, these three pairs of electrodes work as asymmetric cells. (4) AC *versus* AC, which is a symmetric combination.

**Fig. 5:**
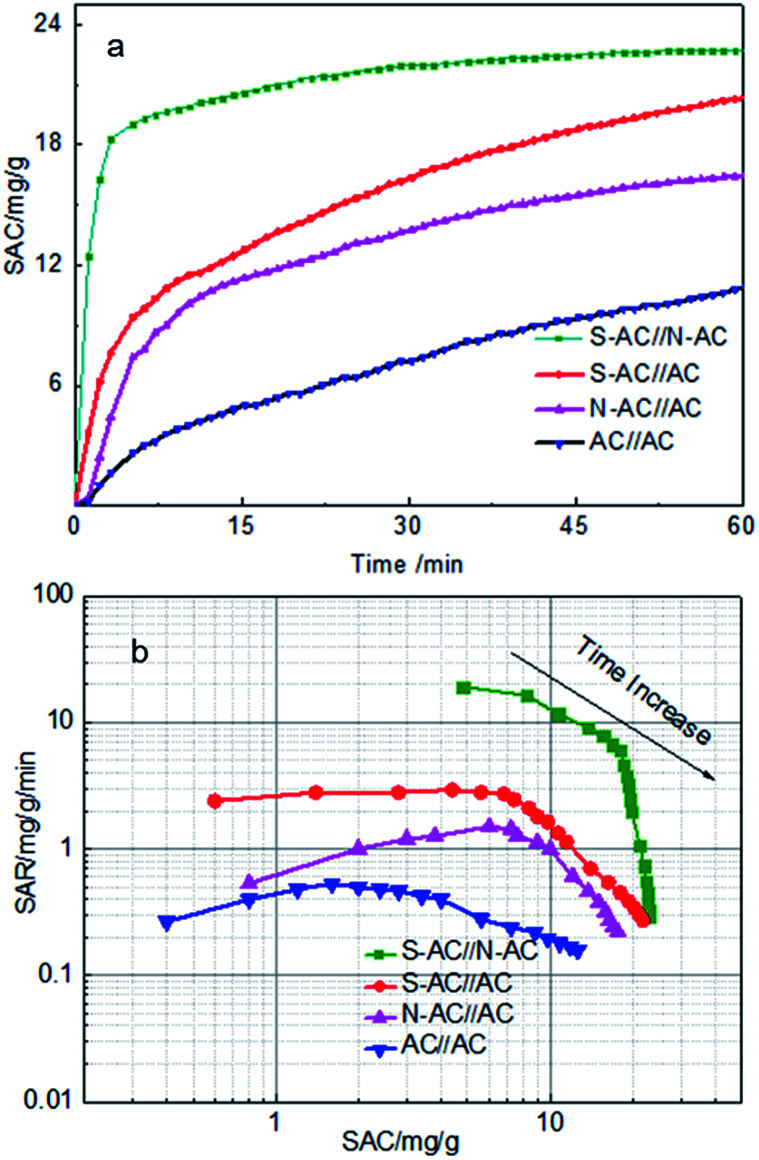
(a) SAC changes *vs.* time curves and (b) Ragone plots of SAR *vs.* SAC in a 1000 mg L^−1^ NaCl solution at 1.4 V with a flow rate of 40 mL min^−1^.

The CDI performance of four different cells were carried out and shown in Fig. 5a. The salt adsorption capacity (SAC) is a useful and insightful performance indicator of the electrodes materials itself and doesn't change with any other cell component under the given experimental conditions.^10^Fig. 5a displays the SAC variation along with the desalination time. Obviously, the SAC of all samples increase rapidly at the beginning 10 min, suggesting that Na^+^ and Cl^−^ are absorbed onto the anode and cathode electrodes as soon as the external voltage is applied. With the time going by, the SAC increase slowly in the 10–60 min, indicating that most of ions have been absorbed onto the electrodes. The SAC keeps nearly smooth after about 60 min, demonstrating that the electrosorption equilibrium time is approximate 60 min.

As calculated, the SAC of asymmetric electrodes were 23.2 mg g^−1^, 20.1 mg g^−1^ and 16.5 mg g^−1^ for the S-AC∥N-AC, S-AC∥AC, N-AC∥AC cells compared with the symmetric AC∥AC cells (11.0 mg g^−1^) at 60 min as the external voltage applied. The corresponding Regone plot SAR (salt adsorption rate) *versus* SAC (according to the eqn (S2) and (S3)†) are provided in Fig. 5b. The plot curves of the S-AC∥N-AC electrodes are in the top and right region compared with AC∥AC electrodes in the down and left region, indicating that the S-AC∥N-AC electrodes have higher SAC and faster SAR. The results can be attributed to the grafted ion-selective –SO_3_^−^ and –NH_3_^+^ functional groups, which can increase the electrostatic interaction force for the counter-ions and repel co-ion to move to the opposite electrode. The hydrophilic –SO_3_^−^ and –NH_3_^+^ groups on the surface increases the electrode's wettability and heighten the interaction between salt solutions and the electrodes' surface.^36^ That is why asymmetric electrode have higher electricsorption performance than symmetric electrodes.

**Fig. 6:**
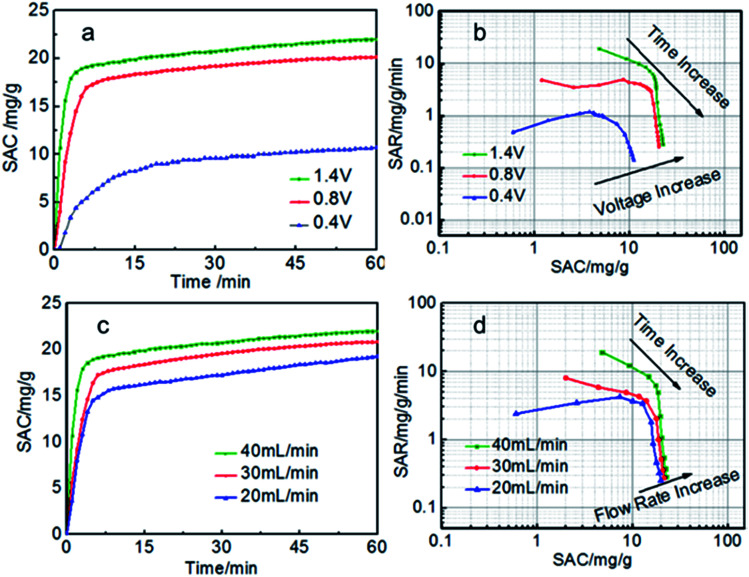
(a, c) SAC curves and (b, d) Ragone plots of SAR *vs.* SAC of the S-AC∥N-AC electrodes in a 1000 mg L^−1^ NaCl solution.

Generally, for the given electrodes material, the CDI performance mainly depends on the operating conditions such as the cell voltage and flow rate.^10,41–43^ The CDI performance of symmetric and asymmetric electrodes at different cell voltages were also investigated (Fig. 6a and b). When the voltage is zero, there is nearly no electro sorption. It can be obviously seen that the electrosorption capacity increase sharply as soon as the external voltage is applied and then reach the electrosorption equilibrium after about 60 min when the voltage increase from 0.4 to 1.4 V. There is no hydrolysis of water observed during the desalination process due to the intrinsic resistance in the whole circuit.^29^ Too higher voltage is not selected because of too higher voltage leading to the hydrolysis of water. The SAC of the S-AC∥N-AC electrodes increased from 8.9 mg g^−1^ to 23.2 mg g^−1^, when the voltage increased from 0.4 V to 1.4 V. However, the symmetric AC∥AC electrodes only increase from 4 mg g^−1^ to 11.0 mg g^−1^. The SAC of asymmetric S-AC∥N-AC electrodes are higher than that of symmetric AC–AC electrodes (Fig. S2a and b†) at any voltage. The higher cell voltage can provide stronger electrostatic interaction which results in a higher salt adsorption.

The experiments at different flow rates were also performed (Fig. 6c and d). The SAC increase with the flow rate increasing from 20 to 40 mL min^−1^. The highest SAC is achieved at flow rate of 40 mL min^−1^ for both S-AC∥N-AC and AC∥AC capacitors. This may be due to that more salt ions can be provided for the adsorption at higher flow rate. The SAC of asymmetric S-AC∥N-AC capacitors at each flow rate are higher than that of symmetric AC∥AC capacitors. All the results indicate that the asymmetric S-AC∥N-AC capacitors have enhanced desalination performance compared with symmetric AC∥AC capacitor (Fig. S2c and d†).

**Fig. 7:**
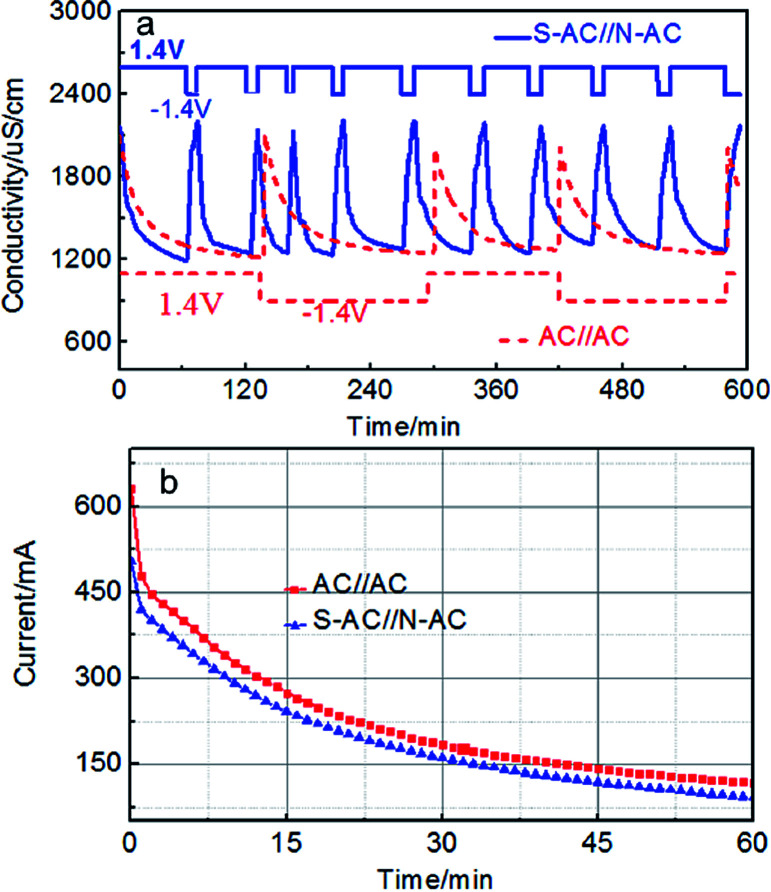
(a) The regeneration curves and (b) current transient of the S-AC∥N-AC and AC∥AC electrodes in a 1000 mg L^−1^ NaCl solution.

The repeated deionization-regeneration experiments of S-AC∥N-AC and AC∥AC capacitors were also evaluated (Fig. 7a). The Na^+^ and Cl^−^ ions are absorbed onto the electrode surface by electrostatic attraction during the charging process. The adsorbed Na^+^, Cl^−^ ions can be effectively and rapidly back to the solution when the applied potential was reversed. As shown in Fig. 7a, the solution conductivity of S-AC∥N-AC capacitors drops more quickly under the applied voltage, and it is more rapidly restored to the initial conductivity once the voltage is reversed compared with AC∥AC capacitors. To finish one deionization-regeneration cycle, it takes about one hour for S-AC∥N-AC capacitors, but two or three hours for AC∥AC capacitors.

The desalination decline of S-AC∥N-AC capacitors did not appear even after 10 cycles. However, the desalination performances tend to decline for AC∥AC capacitors after finishing each deionization-regeneration cycle. The perfect regeneration of the S-AC∥N-AC capacitors can be contributed to the enhanced electrostatic adsorption which successfully prohibits co-ion effect by grafting ion selective functional groups.

The CE is a powerful tool to evaluate charge utilization and energy consumption.^44–51^ The higher CE means the lower energy consumption during the deionization process.^45^ The current response curves of S-AC∥N-AC and AC∥AC capacitors are shown in Fig. 7b. The CE of the S-AC∥N-AC capacitors is calculated to be 0.84 which is higher than that of AC∥AC (0.54) according to the eqn (S4)† and indicating lower energy consumption in this work. It is also higher than most of that reported in literatures (Table 1). This is attributed to following reasons: (i) the expelling of co-ions are blocked and cannot leave the electrode regions through the additional electrostatic adsorption by the ion selective and charged functional groups, and consequently, the charge efficiency of asymmetric electrodes is effectively improved. (ii) The improved surface wettability by grafted ion-selective –SO_3_^−^ and –NH_3_^+^ functional groups is beneficial to the EDL formation and thus ensured the greater and faster desalination. The higher CE means lower energy consumption during this the deionization process.

**Table 1:** Comparison of performance of various asymmetric cells

Asymmetric cells	Applied voltage [V]	Initial NaCl concentration [mg L^−1^]	SAC [mg g^−1^]	CE	Ref.
AC∥F-AC electrodes	1.2	500	16.3	0.7	46
Nafion-AC∥AC	Constant current	∼500	10.8	0.45	47
CX∥modified negative CX	1.2–1.4	234	3.0–5.0	—	35
3DNGR∥3DSGR	1.4	500	13.72	0.85	31
ACF-HNO_3_∥ACF	1.2	500	12.8	0.74	48
Sulphonated-RGO/CFC∥CFC	1.4	1000	160 μmol g^−1^	0.45	49
Sulfonated graphene-CFC∥CFC	1.2	400	9.54	0.425	2
Sulfonated graphene/AC∥aminated graphene/AC	Constant current	500	10.3	0.928	37
AC-QPVP∥AC-HNO_3_	1.2	500	20.6	0.68	50
(COO^2−^)-3DAPGr∥NR_4_^+^-3DAPGr	1.4	300	18.43	0.87	51
S-AC∥N-AC	1.4	1000	23.2	0.84	This work

## 4. Conclusions

In summary, we prepared asymmetric activated carbon electrodes with ion-selective functional groups. The prepared electrodes have higher charge efficiency (0.84) and higher electrosorption capacity (23.2 mg g^−1^) compared to pristine AC electrodes (0.54 of charge efficiency and 11.0 mg g^−1^ of electrosorption capacity) due to the wettability and hydrophilicity effectively enhanced by grafting ion-selective functional groups. Asymmetric AC electrodes show the better cycling stability. These experimental results have demonstrated that the modification of the ion-selective functional groups on the surfaces of AC could reduce the co-ion effects effectively and improve the salt removal efficiency from the solution. This will open the new opportunity to development of capacitive deionization technology for practical desalination.

## Conflicts of interest

There are no conflicts of interest to declare.

## Supplementary Material

RA-008-C7RA10443J-s001
