# Supplementary material for: Ion-selective asymmetric carbon electrodes for enhanced capacitive deionization
Source: RSC Adv. 2018 Jan 11;8(5):2490–7. doi: 10.1039/c7ra10443j (PMC9077380; doi:10.1039/c7ra10443j)
Supplement: RA-008-C7RA10443J-s001 [file RA-008-C7RA10443J-s001.pdf]

Electronic Supplementary Material (ESI) for RSC Advances.
This journal is © The Royal Society of Chemistry 2017

Electronic Supplementary Information (ESI):

Ion-selective Asymmetric Carbon Electrodes for Enhanced Capacitive Deionization

Tingting Yan, Baoxia Xu, Jianping Zhang, Liyi Shi, and Dengsong Zhang*

Research Center of Nano Science and Technology, Shanghai University, Shanghai 200444, China.

Fax: 86 21 66136079; E-mail: dszhang@shu.edu.cn

Characterization

The morphologies were examined by field emission scanning electron microscopy (SEM, JEOL JSM-700F) and powdered samples were dispersed in ethanol by ultrasonication for 10 min in an ultrasonic bath. X-Ray diffraction (XRD) measurements were taken on a Rigaku D/MAX-RB X-ray diffractometer using Cu Kα radiation (40kV, 30 20 mA) and a secondary beam graphite monochromator The Raman spectra were recorded on a spectrometer (JY H800UV) equipped with an optical microscope at room temperature. For excitation, the 633 nm line rom a HeNe ion laser (Spectra Physics) was focused; with an analyzing spot of about 1 mm, on 35 the sample under the microscope. Nitrogen sorption isotherms were measured with an Autosorb-IQ2, Quantachrome Corporation at 77 K. Before the measurements, all samples were degassed overnight at 593 K in a vacuum line. The Brunauer–Emmett–Teller (BET) method was utilized to calculate the specific surface areas and the pore volumes, and the pore size distributions were derived from the desorption branches of the isotherms using nonlocal density functional theory (NLDFT). The X-ray photoelectron spectroscopy (XPS) was recorded on a Perkin-Elmer PHI 5000C ESCA system equipped with a dual X-ray source, using the 45 MgKα (1253.6 eV) anode and a hemispherical energy analyser. The back ground pressure during data acquisition was kept below 10−6 Pa. All binding energies were calibrated using contaminant carbon (C 1s = 284.6 eV) as a reference.

The electrochemical performance of the electrodes was evaluated by cyclic voltammetry (CV), the electrochemical impedance spectroscope (EIS) using a CHI 660D. Galvanostatic charge–discharge was conducted on an automatic

LAND battery test instrument. All the electrochemical properties were measured from a 3-electrode system in a NaCl solution. The specific capacitances were obtained according to the following equation:

$$C = (\int IdV) / 2\text{vD}\Delta\text{Vm} \qquad (1)$$

Herein, the salt adsorption capacity (SAC) of the electrode materials was calculated according to the following equation:

$$SAC = (C_0 - C)V / m \quad (2)$$

where SAC is salt adsorption capacity, C0 and C are the initial and final concentrations, and V is the total volume of the NaCl aqueous solution. Meanwhile, m represents the total mass of the electrodes.

$$SAR = SAC/t \qquad (3)$$

where SAR represents the salt adsorption rate, t stands for the adsorption time and SAC refers to the salt adsorption capacity.

The charge efficiency (Λ) is described according to the following equation:

$$\Lambda = \frac{\Gamma \times F}{\Sigma} \qquad (4)$$

where F is the Faraday constant (96485 C mol-1), Γ is the salt adsorption capacity (mol g-1) and Σ (charge, C g-1) is obtained by integrating the corresponding current.

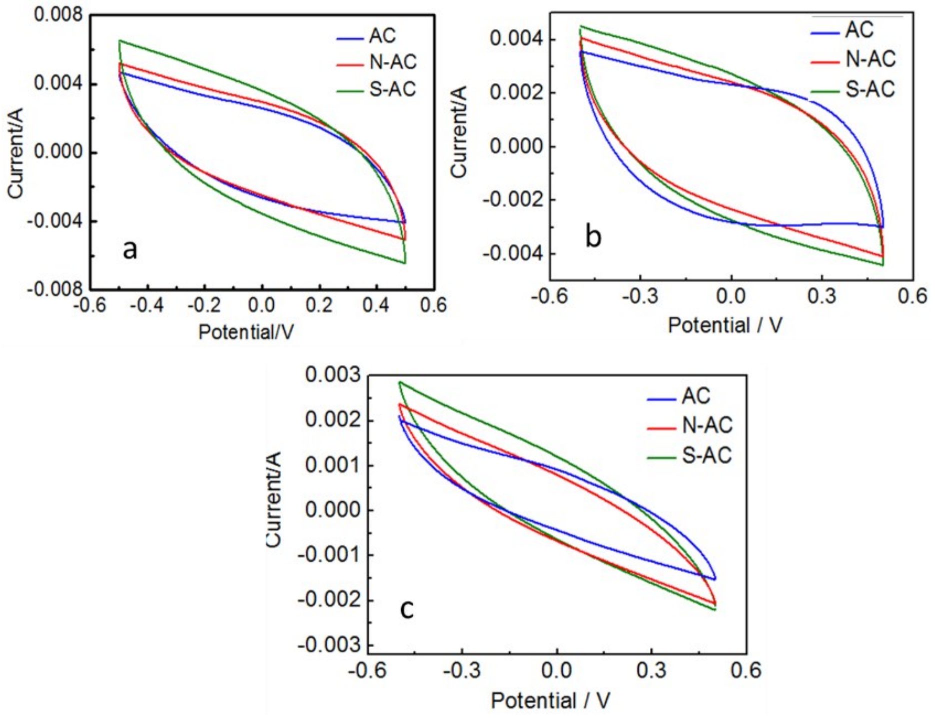

Fig.S1 CV curves of AC, S-AC and N-AC (a) at 10 mV/s in a 0.5 M NaCl solution, (b) at 5 mV/s in a 0.5 M NaCl solution and (c) at at 10 mV/s in a 1000 mg/L NaCl solution

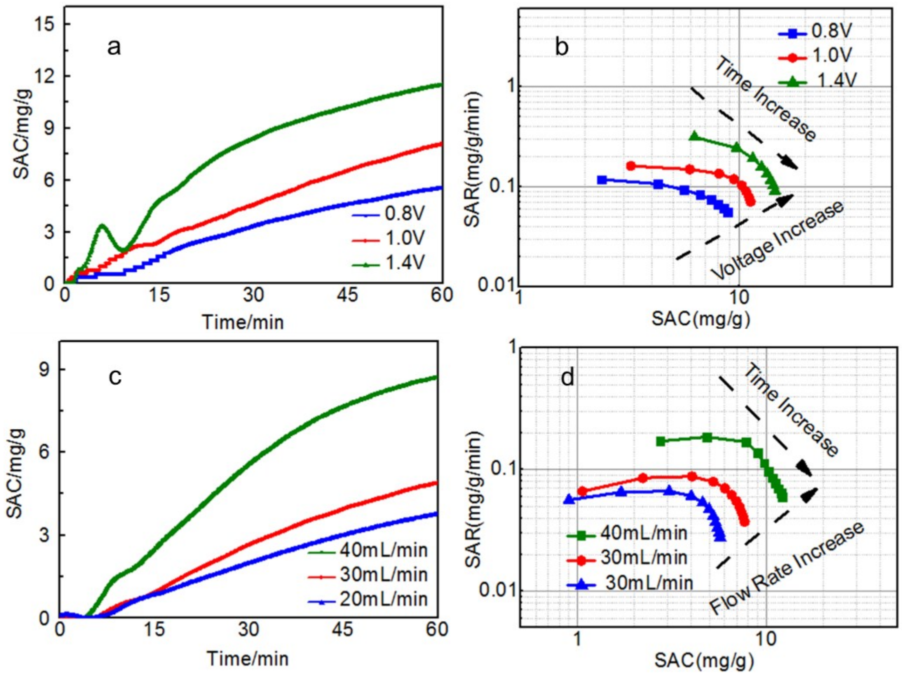

Fig. S2 SAC curves (a, c) and Ragone plots (b, d) of SAR vs SAC of the AC // AC capacitors in a 1000mg/L NaCl solution
